# Biomolecular composition of capping layer and stability of biogenic selenium nanoparticles synthesized by five bacterial species

**DOI:** 10.1111/1751-7915.13666

**Published:** 2020-10-17

**Authors:** Alessandra Bulgarini, Silvia Lampis, Raymond J. Turner, Giovanni Vallini

**Affiliations:** ^1^ Department of Biotechnology University of Verona Strada Le Grazie 15 Verona 37134 Italy; ^2^ Microbial Biochemistry Laboratory Department of Biological Sciences University of Calgary 2500 University Dr. NW Calgary AB T2N 1N4 Canada

## Abstract

Biogenic metal/metalloid nanoparticles of microbial origin retain a functional biomolecular capping layer that confers structural stability. Little is known about the composition of such capping material. In this study, selenium nanoparticles (SeNPs) synthesized by five different bacterial strains underwent comparative analysis with newly proposed protocols for quantifying the concentration of carbohydrates, proteins and lipids present in capping layers. SeNPs were therefore treated with two different detergents to remove portions of the surrounding caps in order to assess the resulting effects. Capping material quantification was carried out along with the measure of parameters such as hydrodynamic diameter, polydispersity and surface charge. SeNPs from the five strains showed differences in their distinct biomolecule ratios. On the other hand, structural changes in the nanoparticles induced by detergents did not correlate with the amounts of capping matrix removed. Thus, the present investigation suggests a hypothesis to describe capping layer composition of the bacterial SeNPs: some biomolecules are bound more strongly than others to the core metalloid matrix, so that the diverse capping layer components differentially contribute to the overall structural characteristics of the nanoparticles. Furthermore, the application of the approach here in combining quantification of cap‐associated biomolecules with the measurement of structural integrity‐related parameters can give the biogenic nanomaterial field useful information to construct a data bank on biogenically synthesized nanostructures.

## Graphical abstract

(i) In this study we propose new protocols for quantifying the concentration of carbohydrates, proteins, and lipids present in capping layers of biogenic selenium nanoparticles. We applied them to compare BioSeNPs produced by five different bacterial strains. (ii) A hypothesis to describe capping layer composition of the bacterial SeNPs is suggested: some biomolecules are bound more strongly than others to the core metalloid matrix, so that the diverse capping layer components differentially contribute to the overall structural characteristics of the nanostructures. (iii) The application of the approach here in combining quantification of cap‐associated biomolecules with the measurement of structural integrity related parameters, can give the biogenic nanomaterial field useful information to construct a data bank on biogenic nanomaterials.

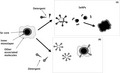

## Introduction

Selenium nanoparticles (SeNPs) of 10–400 nm in size can be produced as spherical aggregates by means of chemically or microbially based protocols (Narayanan and Sakthivel, 2010). These nanomaterials find potential applications in the electronic field, in procedures for bioremediation of heavy metals, as well as for their antioxidant and antimicrobial properties (Fellowes *et al*., 2011; Zonaro *et al*., 2015; Jain *et al*., 2016; Cremonini *et al*., 2016). Selenium nanoparticles can be obtained by chemical synthesis (ChSeNPs), yet those generated through biological process (BioSeNPs) are receiving growing attention, as a variety of microorganisms or microbial consortia have been found to have the capacity to reduce Se‐oxyanions leading to the formation of SeNPs (Kessi, 2006; Butler *et al*., 2012; Lampis *et al*., 2014; Jain *et al*., 2016; Lampis *et al*., 2017; Presentato *et al*., 2018). Additionally, SeNPs can be obtained by exploiting the reducing capability of certain plant extracts or enzymatic preparations towards Se‐oxyanions (Prasad *et al*., 2013).

Selenium nanoparticles generated by microbial species or in the presence of organic ingredients of biological origin in the reaction medium have quite different properties from ChSeNPs due to the interactions of such biomolecules, which associate with the nanoparticles during their synthesis (Zannoni *et al*., 2008; Lenz *et al*., 2011; Jain *et al*., 2015a; Tugarova *et al*., 2018). These biomolecules make up the ‘capping layer’ since they surround the surface of SeNPs. To date, there is no common definition in the literature for this organic cap. Little is known about the specific origin, composition, variability and functional role of such a capping material compared to the uniform cap of chemically synthesized nanomaterials. It is clear though that the properties of SeNPs are greatly influenced by the biomolecules associated with them as it has been shown that once ChSeNPs have been mixed with organic molecules, they change their characteristics (Piacenza *et al*., 2018a).

ChSeNPs can recruit a biological capping layer if exposed to cell extracts or solutions/suspensions of biochemical compounds (Dobias *et al*., 2011). For instance, when chemical synthesis of nanoparticles occurs in the presence of organic molecules – such as polysaccharides, proteins or extracellular polymeric substance (EPS) – these components become part of a capping layer (Dobias *et al*., 2011; Xiao *et al*., 2017). The surrounding organic caps modify reactivity, structural integrity, thermodynamic and chemical stability of ChSeNPs, as well as their antimicrobial and anticancer activity by influencing the uptake of SeNPs by target microorganisms or cancer cells (Stark, 2011). For example, addition of the enzyme alcohol dehydrogenase, previously identified on *E. coli* BioSeNPs, to ChSeNPs caused a decrease in the final size of SeNPs with respect to ChSeNPs synthesized in the absence of this enzyme (Dobias *et al*., 2011). Moreover, ChSeNPs exposed to bovine serum albumin (BSA) or EPS resulted in more spherical and stable NPs compared to untreated ChSeNPs (Jain *et al*., 2016). Stabilizing effects due to capping molecules were also observed in ChSeNPs in the presence of polysaccharides (Zhang *et al*., 2004). It has been hypothesized that the high viscosity of capping molecules may be the reason for their structural integrity and final dimensions. SeNPs formed in the presence of EPS could become trapped in this matrix that then hinders further evolution of aggregation or precipitation dynamics (Xiao *et al*., 2017).

Organic capping has been shown to influence other functional properties of SeNPS. In fact, honey polyphenols associated with ChSeNPs are more efficacious in preventing microbial biofilm formation when compared to uncapped SeNPs or polyphenols alone (Prateeksha *et al*., 2017). SeNP toxicity also varies depending on the nature of the organic components of the capping layer. Chitosan‐coated ChSeNPs exhibited lower toxicity than detergent‐surrounded ChSeNPs (Palomo‐Siguero and Madrid, 2017). Finally, antioxidant properties are even further influenced by the capping layer. EPS‐coated ChSeNPs show a more stringent antioxidant effect than BSA‐coated ChSeNPs (Cheng *et al*., 2017).

Studies on ChSeNPs exposed to or conjugated with defined organic matrices revealed the influence of single molecular components of the capping layer. Nevertheless, little is known about quite complex capping layers occurring in BioSeNPs. Moreover, capping layer formation itself and properties of BioSeNPs are certainly influenced by both the microbial strain used and the culture conditions adopted (Piacenza *et al*., 2018b, 2019).

Biogenic SeNP’s antioxidant, anticancer (Xu *et al*., 2018a) and antimicrobial (Zonaro *et al*., 2017) characteristics are all markedly influenced by their biomolecular capping layer. Modification of the capping layer by procedures such as treatments with detergents can lead to particle aggregation as well as modified antimicrobial properties (Cremonini *et al*., 2018; Xu *et al*., 2018b). As already mentioned for conjugated ChSeNPs, the capping layer contributes even in BioSeNPs to maintain structural integrity of nanoparticles, as a consequence of electrostatic stabilization, allowing SeNPs to remain suspended in aqueous solution due to the induced negative surface charge (Jain *et al*., 2015b; Tugarova and Kamnev, 2017; Xu *et al*., 2018b; Piacenza *et al*., 2019).

Evidence exists that certain organic components are associated with BioSeNPs through interactions seemingly stronger than others, as increasing harshness of detergent treatments selectively remove only portions of the capping layer (Dobias *et al*., 2011). Biomolecules such as proteins, carbohydrates, nucleic acids, humic‐like substances and lipids have all been proposed to take part in the capping layer composition of BioSeNPs (Jain *et al*., 2015a,b; Yang *et al*., 2016; Tugarova *et al*., 2018; Cremonini *et al*., 2018; Xu *et al*., 2018a,b).

Various biophysical tools including energy dispersive X‐ray analysis, Fourier transform infrared spectroscopy, or Raman spectroscopy (Cheng *et al*., 2014; Jain *et al*., 2015a; Xiao *et al*., 2017; Vogel *et al*., 2018; Tugarova *et al*., 2018; Xu *et al*., 2018a), and proteomic approaches (Dobias *et al*., 2011; Lenz *et al*., 2011; Gonzalez‐Gil *et al*., 2016; Zhang *et al*., 2019) have been applied to study the capping layer composition of BioSeNPs. These analytical protocols are well suited for the investigation of a few samples providing targeted information. However, these could be challenging for all laboratories to apply as a routine analysis, particularly to compare a large number of different samples.

Other approaches used to characterize nanomaterials on a routine basis include dynamic light scattering (DLS) analysis, that is a quick and cost‐effective tool. Moreover, parameters such as hydrodynamic diameter (Dh), polydispersity index (PdI) and zeta potential can be quickly measured without destroying samples. DLS and zeta potential analyses are informative of the structural and aggregation integrity of SeNPs. An increase in Dh could be either due to assembly of more Se atoms, addition of more capping material or by aggregation into micro‐clusters. The surface charge of SeNPs (defined as zeta potential) is indicative of the ability the particles have to remain suspended in an aqueous environment (Piacenza *et al*., 2018a). PdI indicates the level of uniform dimensional distribution among NPs.

Selenium nanoparticles biosynthesized by five different environmental bacterial isolates were analysed in this study, with respect to the biomolecular composition of the biological capping layer. A rapid assay protocol was developed for the concentration quantification of capping biomolecules belonging to different classes. The effects on structural integrity of BioSeNPs caused by removing various biomolecules from the capping material through different treatments were then investigated.

## Results

### Biosynthesis of SeNPs by bacterial strains

Five bacterial strains were analysed for resistance to selenite and production of SeNPs. *B. mycoides* SeITE01 and *S. maltophilia* SeITE02 were previously isolated and characterized: SeITE01 is able to grow in the presence of 25mM sodium selenite, SeITE02 in 50 mM (Vallini *et al*., 2005; Di Gregorio *et al*., 2005; Lampis *et al*., 2014; Lampis *et al*., 2017). Newly identified strains R2A, R2D and R1E were isolated from a Se‐polluted soil on 10 mM selenite‐added medium and subsequently tested for Gram stain reaction and for minimum inhibitory concentration (MIC): R1E (Gram‐positive) presented a MIC of 75 mM selenite; R2A and R2D (both Gram‐negative) of 100 mM. Newly identified strains were also tested for SeNPs production: as shown in Fig. S5, Se‐nanospheres are visible alongside bacterial cells after a 24‐h exposition to selenite for R2A and R2D, and 72‐h exposition for R1E.

### Capping layer composition and structural integrity

**Fig. 1:**
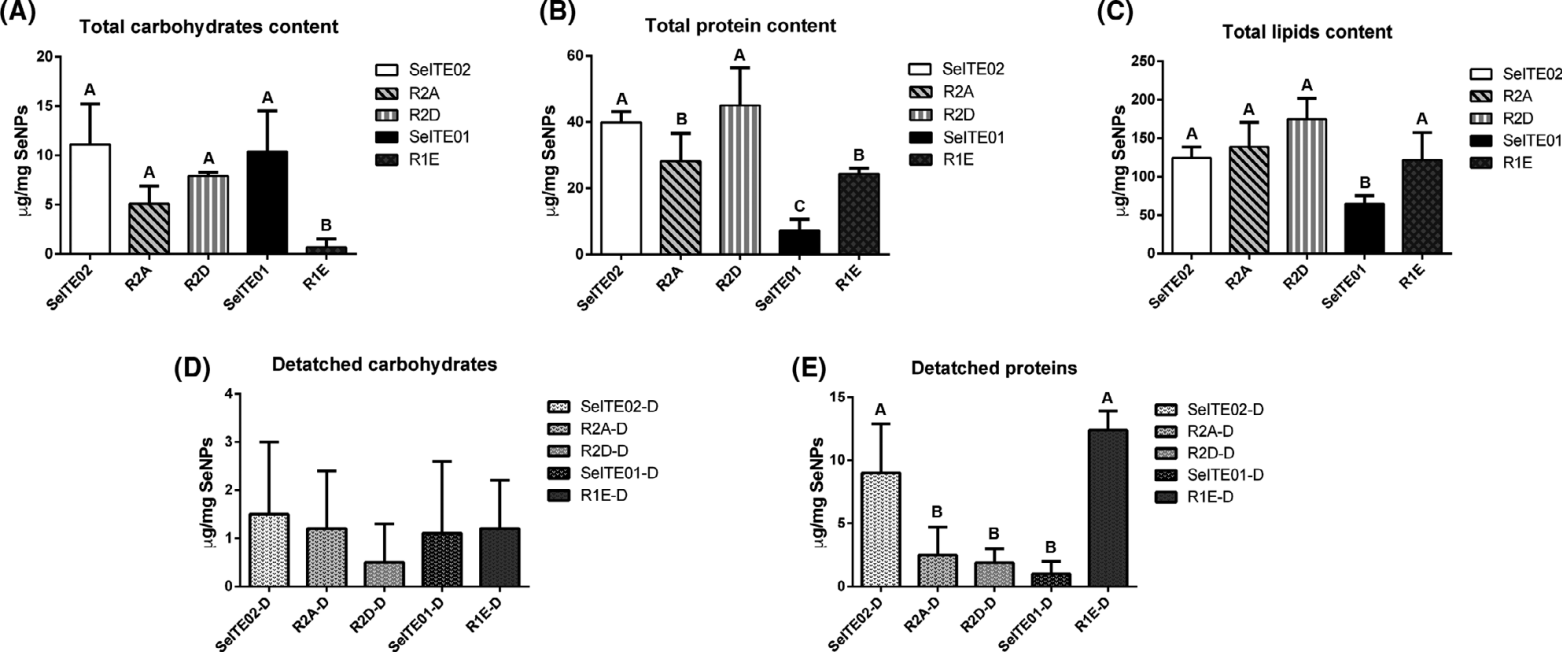
Quantification of carbohydrate (A), protein (B) and lipid (C) content for biosynthesized SeNPs by five bacterial strains SeITE02, R2A, R2D, SeITE01 and R1E directly on SeNPs. Quantification of carbohydrates (D) and proteins (E) dispersed in the supernatant (indicated with ‘strain name‐D’). Letters indicate significant difference: samples with the same letter show no significant difference (ANOVA, *P* < 0.05).

**Fig. 2:**
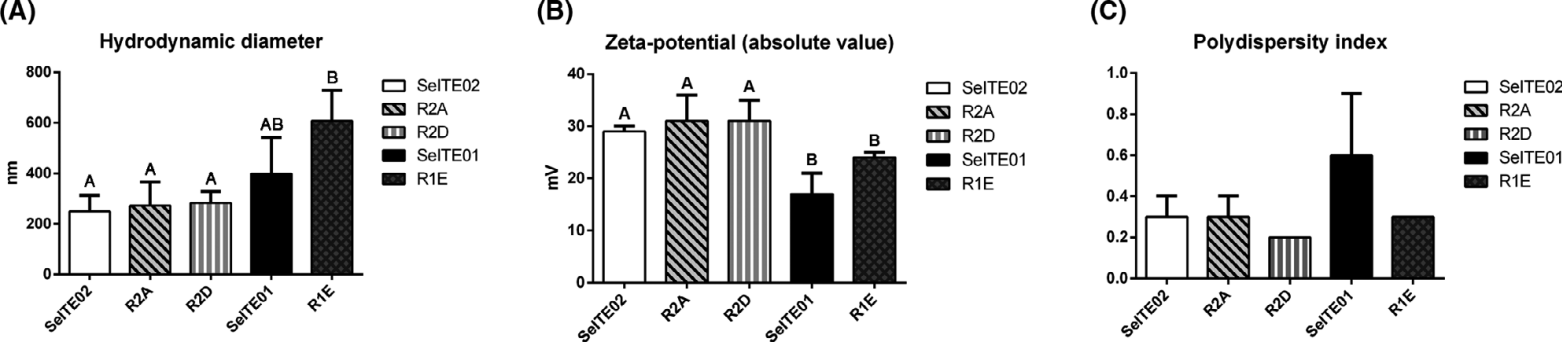
Stability‐associated parameters: hydrodynamic diameter (A), zeta potential (B) and polydispersity (C) for SeNPs synthesized by five bacterial strains SeITE02, R2A, R2D, SeITE01 and R1E. Zeta potential, here reported as absolute value, was negative for all the samples. Letters indicate significant difference: samples with the same letter show no significant difference (ANOVA, *P* < 0.05).

Selenium nanoparticles biosynthesized by SeITE01, SeITE02, R2A, R2D and R1E were extracted and then screened for three different fundamental biochemical compounds potentially associated with the capping layer and for three physical parameters that measure SeNPs structural integrity. Total carbohydrate, protein and lipid contents are shown in Fig. 1, while physical and structural integrity‐related parameters are shown in Fig. 2.

Selenium nanoparticles suspended in sterile water were directly analysed for carbohydrate and protein contents. For the lipid content assay, a lipid extraction step was necessary to collect this material (see methods). Detached material present in the suspension buffer was also analysed after SeNPs removal (indicated in Fig. 1 as ‘strain name‐D’).

Carbohydrate contents show significant difference between SeNP‐attached and SeNP‐detached content for SeNPs by all strains except for R1E (Fig. 1, panels A and D). For protein content, no significant difference was observed for SeITE01 SeNPs between SeNP‐attached and SeNP‐detached proteins (‐D), whereas for SeNPs produced by the other strains, differences were observed between the two samples. SeNP‐attached protein content shows no significant difference between SeITE02 and R2D samples and between R2A and R1E (Fig. 1, panel B). Finally, lipid content significantly differs between SeNPs by SeITE01 and all the other strains (Fig. 1, panel C).

Together with capping components, SeNPs were investigated for size by DLS analysis. Results are shown in Fig. 2. SeITE02, R2A and R2D SeNPs show similar hydrodynamic diameter, significantly differing from R1E. The diameter of SeITE01 SeNPs does not differ significantly from SeNPs by the other strains (Fig. 2, panel A). Zeta potential was found to be negative for all samples, but for convenience of comparison to other panelsit is plotted with absolute values; SeITE01 and R1E SeNPs show similar smaller potentials and were significantly different from the potentials of SeNPs by the three Gram‐negative strains (Fig. 2, panel B). Finally, no significant difference was observed for polydispersity (Fig. 2, panel C). Overall, SeNPs synthesized by Gram‐negative strains seem to be the most stable NPs, with smaller diameters and higher surface charge compared to those produced by the Gram‐positive strains.

### Effects of detergent treatments on the capping layer composition and structural integrity of SeNPs

**Fig. 3:**
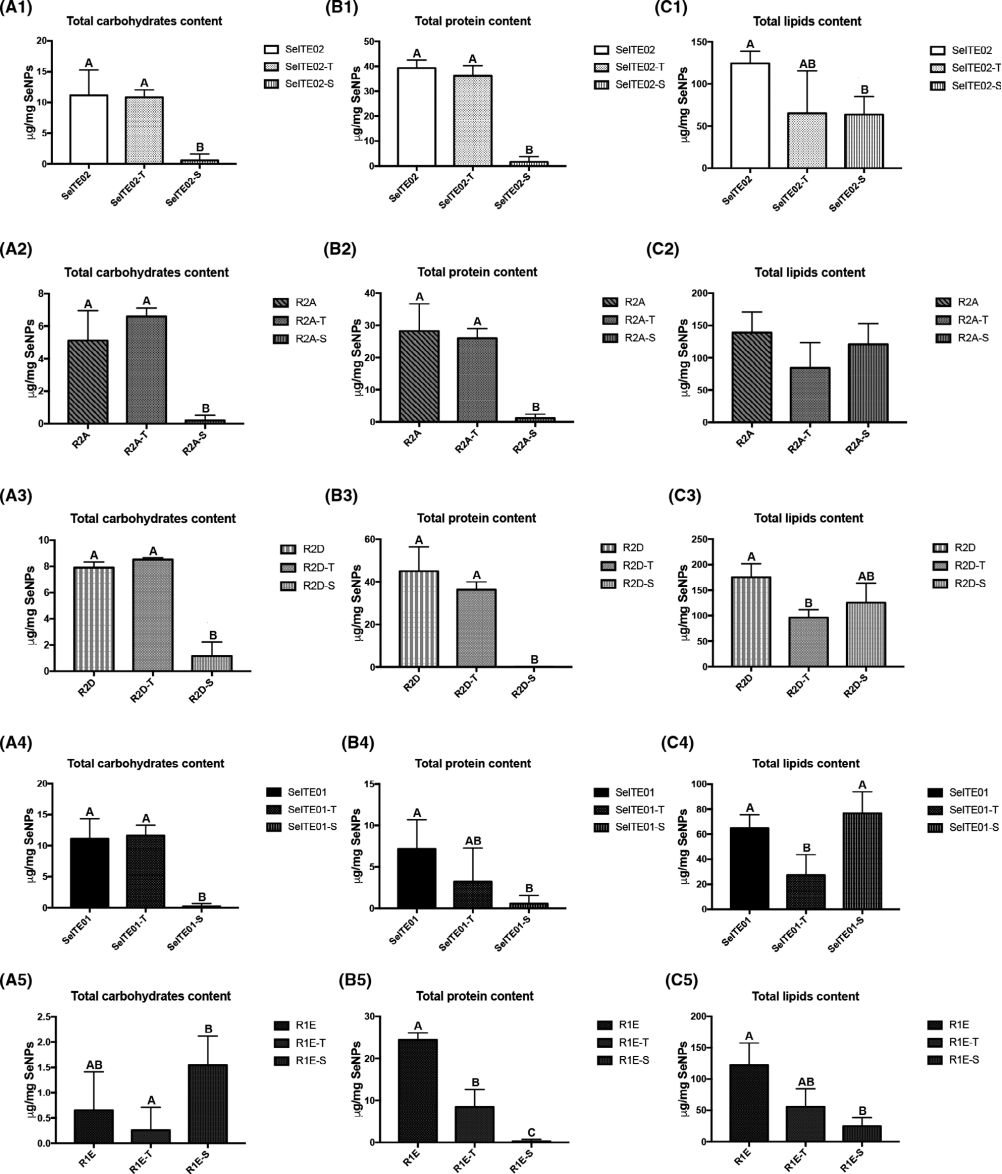
Effect of detergent treatments on biochemical compound levels associated with the biosynthetic SeNP. Quantifications refer to total carbohydrates (column A), proteins (column B) and lipids (column C). Mild treatment with 2% Triton X‐100 is indicated with ‘T’; harsh treatment with 10% SDS at 100°C is indicated with ‘S’. Letters indicate significant difference compared to control: samples with the same letter show no significant difference (unpaired *t*‐test, *P* < 0.05).

Total components and DLS parameters were again assayed after a mild (2% Triton X‐100) or harsh (10% SDS, 100°C) detergent treatment. Such treatments are expected to affect the capping layer composition and the outcomes are shown in Fig. 3. Overall, for Triton treatment, no significant difference was observed between control and treated samples in quantified carbohydrates (column A) or proteins (column B), with the exception of R1E SeNPs protein content (panel B5). On the other hand, significant difference was observed for R2D and SeITE01 lipids contents, which are significantly lowered after the mild Triton treatment (panels C3 and C4).

For SDS treatment, carbohydrate and protein contents were significantly lowered (columns A and B), with the only exception of R1E (panel A5). Lipid content was significantly affected for SeITE02 and R1E (panels C1 and C5).

**Fig. 4:**
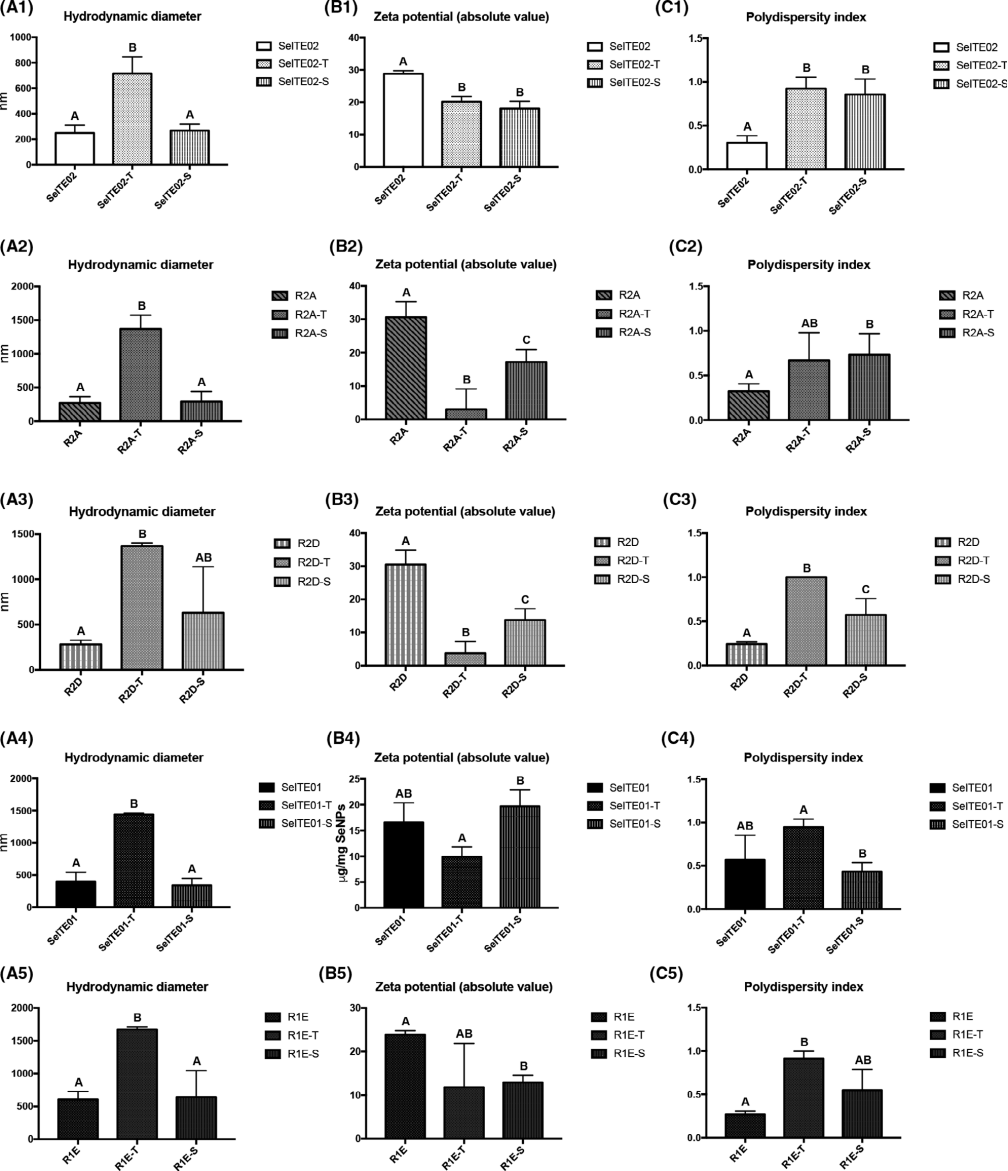
Effect of mild and harsh detergent treatments on stability‐associated parameters from DLS analysis: hydrodynamic diameter (column A), zeta potential (column B) and polydispersity index (column C). Zeta potential, here expressed as absolute value, was negative for all samples. Mild treatment with 2% Triton X‐100 is indicated with ‘T’; harsh treatment with 10% SDS at 100°C is indicated with ‘S’. Letters indicate significant difference compared to control: samples with the same letter show no significant difference (unpaired *t*‐test, *P* < 0.05).

Treatment effects on SeNPs structural integrity are shown in Fig. 4. Overall, for Triton treatment, hydrodynamic diameter significantly increased for all samples (column A). Zeta potential significantly decreased in absolute value for all Gram‐negative samples (panels B1 to B3), while no significant difference was observed for SeITE01 and R1E compared to controls (panels B4 and B5). Finally, polydispersity was significantly higher after Triton treatment for SeITE02, R2D and R1E. All three effects indicate a destabilization of SeNPs, which can be interpreted as particle aggregation (as seen by increasing hydrodynamic diameter).

For SDS treatment, no significant change was observed in hydrodynamic diameter (column A), while zeta potential significantly decreased in absolute value for most samples except for SeITE01 SeNPs (panel B4). Different results were observed for SeNPs polydispersity, which significantly increased after SDS treatment for Gram‐negative samples (panels C1 to C3), while no significant difference was observed for the other strains SeNPs.

### TEM analysis

To further investigate the effect of treatments, SeNPs synthesized by two strains, SeITE01 and SeITE02, were analysed by TEM.

In Fig. S6, SeNPs by SeITE01 are shown: SeNPs can be easily distinguished for standard samples, while for Triton‐treated samples, aggregates can be observed. SDS‐treated samples appear similar to standard ones. In Fig. S7, SeNPs by SeITE02 are shown: differently from the previous samples, Triton‐treated SeNPs were found both unaltered and forming aggregates. Finally, SDS samples were similar to standard ones.

## Discussion

Over the past 10 years, different microorganisms have been found to be able to synthesize metal‐ and metalloid‐based NPs with different properties. Overall, these biogenic NPs show more thermodynamic stability (less potential to aggregate) over time compared to chemically synthesized NPs, which is attributed to attached biological organic molecules originating from the producing strain (Zhang *et al*., 2004; Dobias *et al*., 2011; Jain *et al*., 2015b; Xiao *et al*., 2017; Piacenza *et al*., 2018a). Different molecules have been suggested to be components of BioSeNPs capping layer; however, the composition, structure and role of the capping layer are still under investigation and debated (Piacenza *et al*., 2018a).

Proteomic studies have provided evidence for the presence of proteins in the capping layer (Lenz *et al*., 2011; Gonzalez‐Gil *et al*., 2016). Some authors have also observed that not all proteins attach to the capping layer with the same strength or specificity (Dobias *et al*., 2011). Other molecules found to be part of the capping layer are polysaccharides and EPS‐like material; techniques such as acid–base titration coupled with microscopy and EDXS were utilized to determine chemical groups associated with SeNPs (Jain *et al*., 2015a). Carbohydrates and proteins were confirmed as capping layer components through FTIR (Cheng *et al*., 2017). Finally, the presence of lipids was also observed using Scanning transmission X‐ray microscopy (Yang *et al*., 2016), X‐ray photoelectron spectroscopy (Gonzalez‐Gil *et al*., 2016) or FTIR spectroscopy combining specific absorption bands related to aliphatic chains and to the ester moiety –C(=O)‐O‐C (Kamnev *et al*., 2017; Tugarova *et al*., 2018). Overall, it seems that for most BioSeNPs, the capping layer is composed of EPS‐like molecules (e.g. polysaccharides, proteins). However, all the aforementioned studies analyse SeNPs produced by few or one microorganism or community at a time. Moreover, proteomics and mass spectrometry, EDXS and microscopy techniques like TEM and SEM are time‐consuming and expensive methods. In this study, a quick high‐throughput assay method is proposed in order to collect statistically significant amounts of data to investigate the biological molecular component of BioSeNPs synthesized by different strains using small amounts of sample.

### SeNPs from different bacteria show differences in capping composition

In this study, SeNPs were synthesized by five different bacterial strains showing high levels of resistance to selenite and ability to produce SeNPs: *Stenotrophomonas maltophilia* SeITE02, *Achromobacter* sp. R2A and *Ensifer* sp. R2D, *Bacillus mycoides* SeITE01 and *Lysinibacillus* sp. R1E.

Selenium nanoparticles were extracted from the culture using the octanol/water two‐phase extraction system, that has been extensively used for SeNPs recovery from cell lysates: during the separation of the two phases from the octanol‐lysate mix, SeNPs accumulate in the aqueous phase, while impurities as membranes fragments and cell debris remain in the upper phase (Shakibaie *et al*., 2010; Forootanfar *et al*., 2014; Pouri *et al*., 2017).

**Fig. 5:**
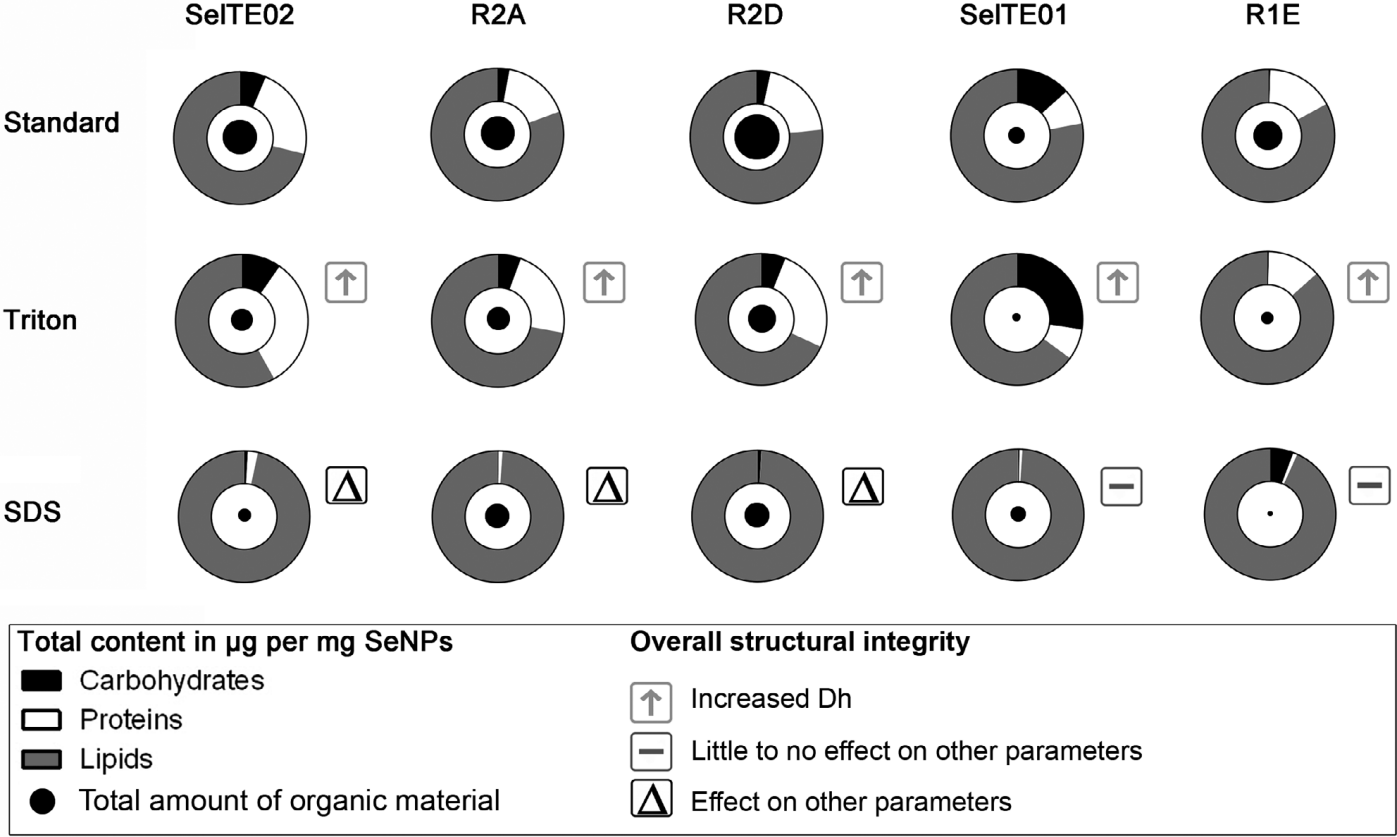
Summary of quantified components as μg mg^−1^ (average) and effect on structural integrity for the five strains. Quantified components are shown in donut charts for all five strains and three treatments. Circles in the centre of charts are scaled to show changes in total organic material content. Overall effect on structural integrity is estimated considering variation in hydrodynamic diameter (Dh), polydispersity (PdI) and surface charge (Z‐pot).

The data collected using the new quantification methods elegantly demonstrated that different types of bacteria produce different NPs with regard to biomolecular composition of their capping layer. The overall quantification of components is summarized in Fig. 5.

Lipids are the major component of the capping layer for SeNPs produced by all five strains, followed by proteins and minor amounts of carbohydrates (with the exception of SeITE01).

For the SeNPs from Gram‐negative strains SeITE02, R2A and R2D, no significant difference was observed in quantity of carbohydrates, proteins and lipids associated, except for the R2A SeNPs having a lower protein content (see also Fig. 1). On the contrary, SeNPs by Gram‐positive strains SeITE01 and R1E show more variability between each other and compared to Gram‐negative samples. Particularly, R1E carbohydrate content is significantly lower than for the other samples, and for SeITE01, carbohydrate‐to‐protein ratio is significantly higher.

Overall, average total material ranges from 228 μg of organic material per mg of particles for R2D to 83 μg mg^−1^ for SeITE01 (Fig. 5, indicated with full circles). Notably, for the SeNPs from the five strains here tested, Gram‐positive samples show a lower total amount of biomolecular material compared to Gram‐negatives. The overall higher hydrodynamic diameter and lower value of zeta potential (Fig. 4) could indicate a lower stabilizing effect of the Gram‐positive capping compared to the Gram‐negative one. However, this is probably not due to the total amount of material, but to the composition and kind of molecules associated with the particles (see below).

It is still too early to define these observations as universal trends for Gram‐positive versus Gram‐negative organisms due to our small sample size, but with more studies analysing biogenic NPs with this approach, a more comprehensive data bank and trends can be established for the field. Such a defined characterization regime could, and should, in fact, also be adapted for other biogenic metal‐based nanomaterials, an issue already noticed for silver nanoparticles (Durán *et al*., 2016).

### Detergent treatments lead to changes in structural integrity by removal of selective capping molecules

Selenium nanoparticles by all the five strains were subsequently treated with detergents through a mild and a harsh treatment aimed to partially remove capping molecules. Since removal of the capping layer has effects on SeNP integrity of the particles size and structure (Piacenza *et al*., 2018a), a comparison between the amount and type of molecules removed and the effect on structural integrity parameters was performed.

Both mild and harsh treatments lead to the removal of part of the capping layer. We found that all samples treated with detergents are characterized by having lower amount of biochemicals compared to the control starting material (Fig. 5). Triton treatment significantly removes lipids for R2D and SeITE01, and proteins for R1E SeNPs. On the other hand, the harsher SDS + heat treatment removes most of carbohydrates and proteins associated with R2A, R2D and SeITE01 SeNPs, and most of proteins and lipids for R1E. Finally, it is significantly effective on all the three analysed components for SeITE02.

Unexpectedly, even if Triton treatment removed less material compared to SDS + heat treatment, the SeNP integrity, as evaluated by the parameters of Dh, PdI and Z‐pot, showed that this treatment led to more aggregation than SDS + heat. This effect was observed for all the five strains SeNPs, regardless of the differences in biological molecule amounts and ratios. Triton treatment led to charge integrity loss and aggregation into larger particles. On the other hand, SDS + heat treatment had differing effects on individual parameters. However, the overall structural integrity of SDS + heat treated samples was still comparable to the untreated samples (especially regarding Dh). Notably, effects of SDS + heat treatment on parameters other than Dh were mainly observed for Gram‐negative samples, while the same treatment is less effective for Gram‐positive SeNPs, causing just a decrease in zeta potential value for R1E (Fig. 5).

Finally, as shown in results, TEM could be useful to further investigate samples considered particularly interesting after the screening protocol, and possibly complementing the DLS analysis. This is an example of further investigations aimed to completely characterize SeNPs with TEM analysis or qualitative techniques such as FTIR and Raman spectroscopies. In fact, this newly developed protocol allows one to collect quantitative data and indications about structural integrity. On the other hand, molecule identification before and after treatments, SeNP diameter and Se content can only be obtained by subsequent analyses.

Here, the use of TEM confirmed the DLS data for Gram‐positive SeITE01 (Fig. S6), as Triton‐treated samples were less stable, while SDS‐treated samples were similar to the untreated (standard) in both DLS and TEM analyses. For Gram‐negative SeITE02 (Fig. S7), mixed results were obtained for Triton‐treated samples by TEM analysis, matching with high polydispersity and Dh shown in DLS analysis. While for SDS‐treated samples polydispersity was also high, no significant difference was observed in Dh compared to standard samples (see Fig. 4).

### Development of a high‐throughput routine screening protocol to compare capping composition and structural integrity of SeNPs

In this study, an assay protocol was optimized for quantification of three biochemical components of the capping layer, in order to compare SeNPs synthesized by different microorganisms and differently treated with biomolecule disruptors such as detergent. The quantification of three biochemical classes (carbohydrates, proteins and lipids) was coupled with dynamic light scattering (DLS) and zeta potential analysis to assess the SeNPs structural integrity. The established protocol used here is facile, quick, cost‐effective and can be used effectively on a large number of samples with high diversity.

Quantification approaches have previously been applied in literature to carbohydrates and proteins. Carbohydrates have been previously quantified using the phenol‐sulfuric acid method (Cheng *et al*., 2017; Xu *et al*., 2018b) or the anthrone method (Cremonini *et al*., 2018), while bicinchoninic acid assay (BCA) is a very sensitive assay for protein quantification (Cheng *et al*., 2017). Lowry assay has also been used to quantify proteins (Cremonini *et al*., 2018; Xu *et al*., 2018b). However, carbohydrate and protein quantification is often performed after a step of stripping away such material from SeNPs. Still some molecules that were more strictly bound to the particles could not be separated and thus included in the final quantification.

Quantification of SeNPs is a challenge. BioSeNPs extracted from cultures are not dissolved in the liquid media, but form a colloidal suspension. Studies so far have quantified SeNPs as ml of BioSeNPs suspension or to total Se content in such suspensions. This method is useful for comparison between samples; however, it is not possible to distinguish between molecules actually associated with BioSeNPs and molecules dissolved in the suspension buffer, and no study so far has attempted to quantify components directly on BioSeNPs. Here microtiter plate assays were developed for quantification of components (carbohydrates, proteins and lipids) directly on BioSeNPs. Assays were considered for parameters of: sensitivity, speed of assay, cost‐effectiveness and ability to compare multiple samples at the same time. Moreover, the assays allowed quantification of the biochemical components to the mass amount of starting NP material (mg of SeNPs). Sensitivity was achieved at μg level, making it possible to quantify components from 0.3 to 0.6 mg SeNPs per sample. Also, microplate assays proved to be cost‐effective, requiring μl‐range volumes of inexpensive reagents. Depending on the sample, molecules used for the standard calibration curves can be substituted. Here, glucose, fructose, galactose, and BSA were used for total carbohydrate and protein quantification (for calibration curve linearity ranges, see Supplemental material, Figs S1–S4). Oleic acid was chosen as a standard for lipids quantification, being a typical fatty acid found in relatively high abundance in membrane phospholipids of bacteria and also a reasonably inexpensive reagent compared to possible phospholipids standards (e.g. POPC or POPE). Unfortunately, this assay does not detect saturated fatty acid content and thus would be an underestimation of total lipid. On the other hand, lipid content and saturation levels may change under selenium stress and NP synthesis, and of course there would be bias on what type of lipid and fatty acid is associated in the cap of the SeNPs. Regardless, the assay is very suitable as the goal is to understand the relative abundances of the three biomolecules classes associated with the biogenic NPs in a quick easy inexpensive high‐throughput assay.

For the physical measurements, parameters such as hydrodynamic diameter (Dh), polydispersity index (PdI) and zeta potential (Z‐pot) were used as they are all informative of SeNP structural integrity, which is often interpreted in how stable the NPs will be in the future. Hydrodynamic diameter can be considered as an indicator of structural integrity, as an increase in this parameter after a treatment is typically the result of NP aggregation into larger micron sized clusters. Notably, Dh analysis through DLS can only be used for spherical SeNPs analysis, not being suitable for rods or cubic NPs. It should be also considered that Dh does not correspond to SeNPs dimension, as the Se core may scatter differently than the capping layer and subsequent loose layer. Se core diameter can be evaluated by electron microscopy analysis, but this is not amenable to a fast routine screening protocol, and could be applied to specific samples after the screening tools used here. On the other hand, the allotropic modifications in the Se core and the bioorganic composition of the capping layer could be assessed by combining Raman and FTIR spectroscopies, respectively, in isolated SeNPs (Vogel *et al*., 2018; Tugarova *et al*., 2018; Tugarova *et al*., 2020; Fischer *et al*., 2020). The NP surface charge (indicated by zeta potential) is indicative of the ability to remain resuspended in aqueous solution. PdI indicates the distribution of SeNP dimensions, giving an idea of the size distribution variance. Thus, here the combination of these methods gives an overview of the SeNP integrity after different treatments. We also complemented this with TEM to get visual confirmation of the degree of aggregation stability.

### Capping layer nature of SeNPs

High‐throughput screening performed in the present study has evidenced differences between SeNPs generated by Gram‐negative and Gram‐positive strains. Overall, harsh detergent treatments seem to be more effective in removing molecules from the capping layer than milder treatments. Yet, overall, structural integrity‐related DLS parameters appear decidedly influenced by the milder treatment, with a significant decrease of surface charge and a significant increase in dimension and polydispersity of SeNPs, which is in agreement with the dogma of NP formation and stability (reviewed by Piacenza *et al*., 2018a). This is revealed by the increased size and aggregation observed in TEM images of SeNPs from samples evaluated this time.

**Fig. 6:**
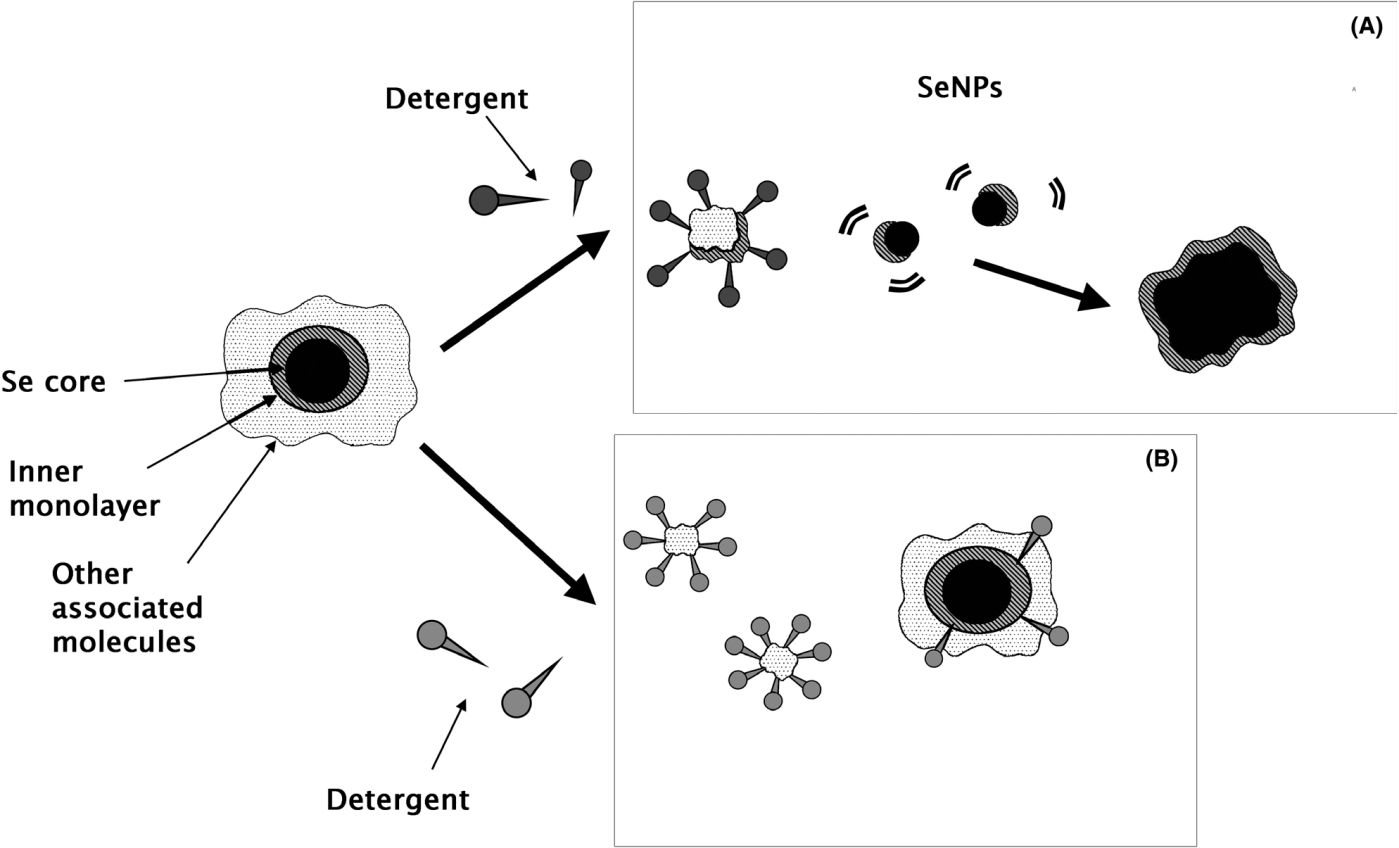
Hypothesis on capping layer structure as composed by two sub‐layers and effect of detergent treatments: capping layer is composed of an inner monolayer of strongly bound molecules and an outer layer of other molecules weakly associated with Se core or with the inner monolayer. This outer layer could be in equilibrium with the resuspension environment. Different detergents could damage the inner layer or interact with the outer molecules. When the inner layer is damaged (even if it cannot be totally detached), it results in SeNP destabilization and aggregation (A). Alternatively, detergents could remove part of the outer molecules, leading to minor destabilization, or also integrate in the outer layer, stabilizing the structure (B).

In Fig. 6, a hypothesis is made for the capping layer structure and potential dynamic equilibrium of biomolecules based on the response to treatments adopted. Since the capping layer cannot be completely removed even after harsh treatments, it can be assumed that some biomolecules are strongly bound to the SeNP inner core. In our hypothesis, such biomolecules would constitute an ‘internal layer’ on Se atom surface of the nanoparticles. On the other hand, some biomolecules can be removed by milder treatments and thus easily detached from the SeNPs. We consider that these molecules constitute an outer layer and are more weakly bound to the inner layer. It is likely that the biomolecules in this layer are in an equilibrium with the molecules in the bulk media. The nanoparticle theory defines the capping layer to provide a stabilizing effect on NPs; however, we do not see a correlation between structural integrity and the amount of material removed. Hence, it can be hypothesized that the loss of any structural integrity is more linked to the type of biomolecule removed.

Here, different detergents can either interact or influence the dissociation with the inner layer molecules (Fig. 6, panel A), or with the outer layer molecules (Fig. 6, panel B). In the first case, regardless of the number of molecules removed from the outer layer, detergents remove part of the inner layer, leading to SeNP aggregation. In the second case, only molecules of the outer layer are removed, while the inner layer is not influenced. This can lead to destabilization, but with a minor effect compared to first case: SeNPs can aggregate, but remain in the nm‐range. Another hypothesis is that a detergent‐like SDS‐ may bind, exchange out and replace other molecules in the outer layer, becoming part of the capping layer and thus changing the stability of the NPs. The last hypothesis could be applied to our samples: despite the amount of material removed after SDS‐heat treatment and the prevalence of lipids (which consist of neutral and non‐ionizable molecules) as the main component (Fig. 5), the Z‐potential is still negative. This could be caused by the incorporation of negatively charged detergent SDS in the outer layer (Fig. 6, panel B).

A goal in nanomaterial technologies is to have unique functionalities with excellent stability. Stability is a function of both thermodynamic and kinetic variables that require careful and full evaluation of various factors, including but not limited to: temperature, chemicals, pH, ionic strength, counter ions, UV or other light damage, redox, and mechanical shear forces. Using the facile high‐throughput approach presented here provides a step towards future studies, where one can evaluate how their biochemical ratio composition affects their given biogenic nanomaterial stabilities and functionalities, whatever they may be.

## Experimental procedures

### Bacterial strains

*Bacillus mycoides* SeITE01 and *Stenotrophomonas maltophilia* SeITE02 have been isolated from the rhizosphere of Se hyperaccumulator plant *Astragalus bisulcatus* as described in Vallini and colleagues (2005) and Di Gregorio and colleagues (2005). R2A, R2D and R1E bacterial strains were isolated from a selenium‐contaminated soil through enrichment cultures in liquid R2A medium (Thermo Fisher, Milan, Italy) with added 10 mM sodium selenite (Sigma‐Aldrich, Milan, Italy). Minimum inhibitory concentration (MIC) was determined by cultivation over a range of Na_2_SeO_3_ concentrations from 5 to 150 mM. For strain identification, 16S rRNA gene was sequenced, and sequencing data were analysed by means of EZCloud system (Yoon *et al*., 2017). Strains were identified by 16S rRNA sequencing: R2A shows identity of 99% to *Achromobacter* sp.; R2D of 98% to *Ensifer* sp.; and R1E of 99% to *Lysinibacillus* sp.

### Biosynthesis of SeNPs and extraction protocol

Bacterial cells were grown aerobically in 400 μl of Nutrient broth medium (Thermo Fisher) with added sodium selenite (0.5 mM for SeITE02; 2 mM for R2D and SeITE01; 5 mM for R2A and R1E), in 1 l flasks on a rotary shaker (150 rpm) at 27°C. SeNPs were extracted after 24 h for SeITE02, R2A, R2D and SeITE01; after 72 h for R1E. Cultures were centrifuged for 10 min at 12 000 *g* in a Sorvall RC‐5C Plus centrifuge, SS‐34 rotor in 50 ml Falcon tubes, and washed twice with 30 ml of 0.9% NaCl for each tube. Pelleted cells were collected and resuspended in 20 ml ice cold 1.5 M Tris‐HCl pH 7.4, splitted in 5 ml aliquots and sonicated in 15 ml Falcon tubes with ultrasonic processor UP50H (Dr. Hielscher GmbH) for 3.5 min alternating sonication and rest in ice. Debris was discarded after centrifugation at 4300 *g*, 4°C for 20 min in a Sorvall Super T21 centrifuge, SL‐50T rotor. Supernatant containing SeNPs was mixed with 1‐octanol (Sigma‐Aldrich): 2 ml of 1‐octanol for each 5 ml lysate aliquot. The mixture was stirred and centrifuged for 5 min at 480 *g*, and finally incubated overnight at 4°C. SeNPs were pelleted from aqueous phase by centrifuging at 18 000 *g* for 20 min, washed once and resuspended in sterile water. SeNPs were collected in Eppendorf tubes, dried for 1–2 h under chemical hood at room T and subsequently quantified by dry weight and stored in sterile water at 4°C.

For detached material sample preparation, SeNPs were precipitated at 16 000 *g*, 20 min. Supernatant containing detached material was collected and analysed as a sample, while SeNP pellet was resuspended in sterile water for subsequent analyses.

### Detergent treatments of SeNPs

Selenium nanoparticles were pelleted at 16 000 *g* for 30 min. Detergent treatments were carried out as follows: for mild treatment, 500 μl of 2% Triton X‐100 was added to the microfuge tube containing SeNP sample. Mixture was incubated on a rotary shaker for 20 min at 27°C. For harsh treatment, 1 ml of 10% sodium dodecyl sulfate (SDS) was added and the mixture was incubated at 100°C for 30 min. Treated SeNPs were finally recovered by centrifugation at 18 000 *g* for 20 min, washed twice and resuspended in sterile water.

### Dynamic Light Scattering (DLS) analysis

Parameters were measured using Malvern Zetasizer Nano Series instrument (Milan, Italy) (software provided by Malvern) as follows: all samples were resuspended in sterile water and transferred to disposable cuvettes (10 mm path length) for Hydrodynamic diameter (Dh) and polydispersity index (PdI) analyses. Zeta potential was measured in sterile water using Folded Capillary Zeta Cell (Malvern).

### Total carbohydrates content assay

For total carbohydrates quantification, a microplate assay protocol was optimized from Masuko and colleagues (2005). A 1:1:1 D(+)‐glucose (Sigma‐Aldrich), D(−)‐fructose (Honeywell‐Fluka, Milan, Italy) and D(+)‐galactose (Honeywell‐Fluka) mixture (called GFG solution) was used to build a calibration curve (0.2, 0.5, 1, 5, 10 μg μl^−1^; Chow and Landhäusser, 2004). A total of 50 μl of GFG solution, samples and blanks were added in a 96‐well plate and background absorbance was read at 490 nm. Chemically synthesized SeNPs (Lin and Wang, 2005) were added to calibration curve to match samples background absorbance. If necessary, more calibration curves were added to the 96‐well plate to match all samples. For quantification, 150 μl of pure (95–97%) sulfuric acid, quickly followed by 30 μl of 2% phenol (in distilled water), was added. Microplate was heated at 90°C for 5 min in a static water bath and then cooled at room T for 5 min. After 18 h, absorbance was measured at 490 nm. Each sample was quantified referring to the corresponding calibration curve.

### Total protein content assay

For total protein content quantification, a protocol was optimized from Minamide and Bamburg (1990). 1x1 cm squares were pencil‐drawn on a Whatman paper sheet and then spotted with 8 μl of samples. Bovine serum albumin (Sigma‐Aldrich) was used as a standard to build the calibration curve (0.016, 0.03, 0.08, 0.3, 0.5, 1, 2 μM). The sheet was then rinsed in absolute methanol and air‐dried under a chemical hood. Protein staining was performed placing the sheet in a 0.5% Coomassie Brilliant Blue G solution in 7% acetic acid and gently agitating for 30 min. Following, the sheet was destained in 7% acetic acid for 30 min to 3 h and air‐dried. Squares were cut and mixed with 1 ml 66% methanol, 33% water and 1% ammonium hydroxide solution to extract Coomassie Brilliant Blue still bound to proteins. Samples were stirred twice on a vortex with a 5 min recovery at 21°C; then, 200 μl of each sample and calibration curve solutions were transferred to a 96‐well plate, and their absorbance was read at 595 nm.

### Total lipid content assay

For total lipid content quantification, a protocol was optimized from Cheng and colleagues (2011), using oleic acid (Alfa Aesar) as calibration standard. Lipids were extracted from SeNPs as follows: SeNPs were pelleted, and supernatant was discarded. Chloroform:methanol 2:1 extraction buffer was added, and the mixture was shaken at 150 rpm for 30 min. SeNPs were pelleted at 18 000 *g* for 20 min and again resuspended in extraction buffer and shaken. Finally, pellet was discarded and chloroform:methanol solution containing the extracted lipids was pipetted in a 96‐well plate under chemical hood. For calibration curve, different quantities of oleic acid were solubilized in chloroform:methanol 2:1 solution (final quantities: 0.78, 2, 5, 10, 30, 70, 100 μg well^−1^). After solvent evaporation, extracted lipids remained in the wells: 100 μl of pure (95–97%) sulfuric acid was immediately added before the lipids dried. Samples were incubated for 20 min at 90°C in a static water bath and immediately cooled for 2 min in ice water. Once the microplate was cold, 50 μl of 0.2 mg ml^−1^ vanillin in 17% phosphoric acid was added. After 18 h, absorbance was measured at 540 nm.

All samples were analysed as follows: three different SeNP batches for each strain and three replicas for each batch. Finally, ANOVA was performed to detect significant difference (*P* < 0.05).

### TEM analysis

TEM analyses were carried out on both BioSeNPs and bacterial cultures exposed to selenite. In the case of BioSeNPs, both standard and treated SeNPs synthesized by SeITE01 and SeITE02 were analysed. On the other hand, bacterial cultures of R2A, R2D and R1E exposed to selenite were sampled and observed after 24 and 72 h of incubation. For each sample, 5 μl was spotted on CF300‐Cu‐Carbon Film Copper grids (CliniSciences, Guidonia Montecelio, Italy) and air‐dried for 24h. Samples were directly observed with Philips CM‐100 electron microscope.

## Conflict of interest

The authors have no conflict of interest to declare.

## Supporting information


**Fig. S1.** Linearity range tested for carbohydrates assay: correlation of carbohydrates concentration with Abs490 using a glucose:fructose:galactose 1:1:1 solution (GFG solution). Correlation is non linear below 0.098 μM GFG (A); linearity ranges from 0.195 μM to 12.5 μM (B). Test was conducted three times (Test 1 to 3).
**Fig. S2.** Calibration curves tested for carbohydrates assay: GFG solution added with chemical SeNPs to match biogenic SeNPs samples typical interference values. All curves maintain linearity between 0.195 μM and 10 μM GFG.
**Fig. S3.** Linearity range tested for proteins assay: correlation of proteins concentration with Abs595 using a bovine serum albumine (BSA) solution. Correlation is non linear below 0.008 μg/μl BSA (A); linearity ranges from 0.016 μg/μl to 2 μg/μl (B). Test was conducted three times (Test 1 to 3).
**Fig. S4.** Linearity range tested for lipids assay: correlation of lipids concentration with Abs540 using a oleic acid solution. Correlation is non linear below 0.39 μg/well oleic acid (A); linearity ranges from 0.78 μg/well to 100 μg/well (B). Above 100 μg/well, linearity is not maintained (C). Test was conducted three times (Test 1 to 3).
**Fig. S5.** Cultures of bacteria in Nutrient medium: R2A: control (A) and after 24 h exposition to selenite. R2D: control (C) and after 24 h exposition to selenite (D). R1E: control (E) and after 72 h exposition to selenite (D). SeNPs are clearly visible in all exposed cultures (B, D, F).
**Fig. S6.** SeNPs synthesized by *B. mycoides* SeITE01: A, B: standard sample. C, D: sample after detergent treatment with 2% Triton X‐100, aggregates are visible as darker clusters. E, F: sample after detergent treatment with 10% SDS.
**Fig. S7.** SeNPs synthesized by *S. maltophilia* SeITE02. A, B: standard sample. C: sample after detergent treatment with 2% Triton X‐100, here SeNPs have not aggregated. D: sample after detergent treatment with 2% Triton X‐100: this is a representative figure of the aggregates which were visible throughout the sample; this correlates well with the measurement of a high Polydispersity (0.9). E, F: sample after detergent treatment with 10% SDS.
