# Supplementary material for: Biomolecular composition of capping layer and stability of biogenic selenium nanoparticles synthesized by five bacterial species
Source: Microb Biotechnol. 2020 Oct 17;14(1):198–212. doi: 10.1111/1751-7915.13666 (PMC7888468; doi:10.1111/1751-7915.13666)
Supplement: Supplementary file 1 — Fig. S1. Linearity range tested for carbohydrates assay: correlation of carbohydrates concentration with Abs490 using a glucose:fructose:galactose 1:1:1 solution (GFG solution). Correlation is non linear below 0.098 μM GFG (A); linearity ranges from 0.195 μM to 12.5 μM (B). Test was conducted three times (Test 1 to 3). Fig. S2. Calibration curves tested for carbohydrates assay: GFG solution added with chemical SeNPs to match biogenic SeNPs samples typical interference values. All curves maintain linearity between 0.195 μM and 10 μM GFG. Fig. S3. Linearity range tested for proteins assay: correlation of proteins concentration with Abs595 using a bovine serum albumine (BSA) solution. Correlation is non linear below 0.008 μg/μl BSA (A); linearity ranges from 0.016 μg/μl to 2 μg/μl (B). Test was conducted three times (Test 1 to 3). Fig. S4. Linearity range tested for lipids assay: correlation of lipids concentration with Abs540 using a oleic acid solution. Correlation is non linear below 0.39 μg/well oleic acid (A); linearity ranges from 0.78 μg/well to 100 μg/well (B). Above 100 μg/well, linearity is not maintained (C). Test was conducted three times (Test 1 to 3). Fig. S5. Cultures of bacteria in Nutrient medium: R2A: control (A) and after 24 h exposition to selenite. R2D: control (C) and after 24 h exposition to selenite (D). R1E: control (E) and after 72 h exposition to selenite (D). SeNPs are clearly visible in all exposed cultures (B, D, F). Fig. S6. SeNPs synthesized by B. mycoides SeITE01: A, B: standard sample. C, D: sample after detergent treatment with 2% Triton X‐100, aggregates are visible as darker clusters. E, F: sample after detergent treatment with 10% SDS. Fig. S7. SeNPs synthesized by S. maltophilia SeITE02. A, B: standard sample. C: sample after detergent treatment with 2% Triton X‐100, here SeNPs have not aggregated. D: sample after detergent treatment with 2% Triton X‐100: this is a representative figure of the aggregates which were visible through [file MBT2-14-198-s001.pdf]

Biomolecular composition of capping layer and stability of biogenic selenium nanoparticles synthesized by five bacterial species.

Alessandra Bulgarini[1,2], Silvia Lampis[1], Raymond J. Turner[2*†] and Giovanni Vallini[1†]*

Supplementary material

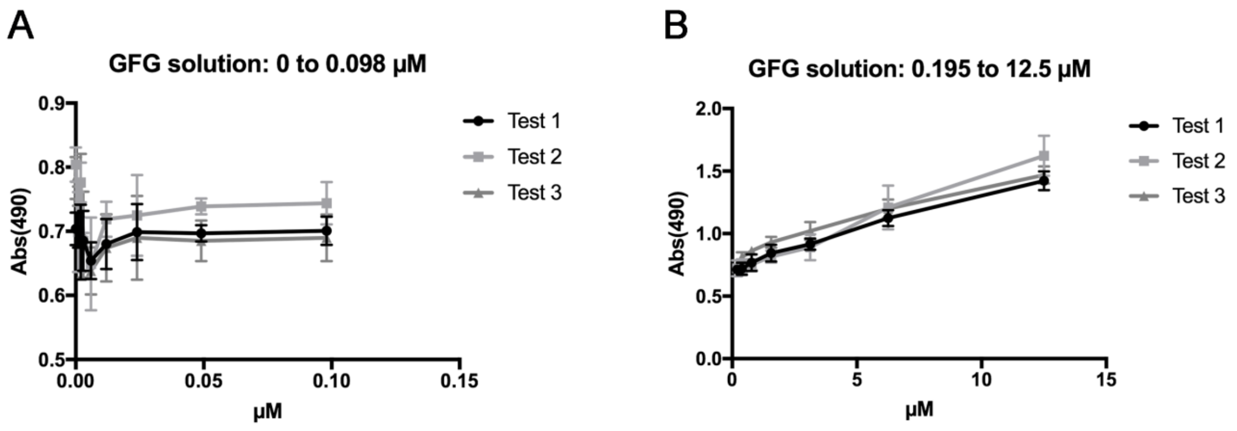

FIGURE 1S
Linearity range tested for carbohydrates assay: correlation of carbohydrates concentration with Abs_{490} using a glucose:fructose:galactose 1:1:1 solution (GFG solution). Correlation is non linear below 0.098 µM GFG (A); linearity ranges from 0.195 µM to 12.5 µM (B). Test was conducted three times (Test 1 to 3).

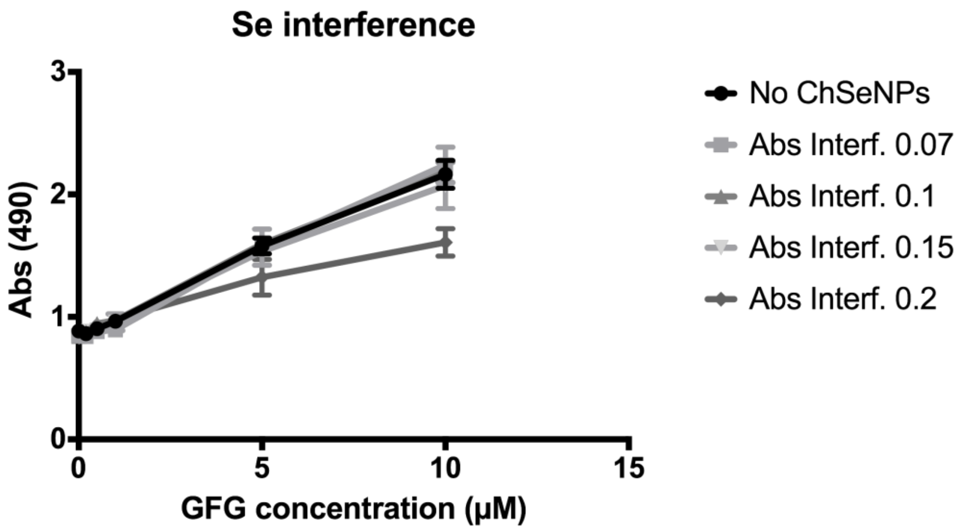

FIGURE 2S

Calibration curves tested for carbohydrates assay: GFG solution added with chemical SeNPs to match biogenic SeNPs samples typical interference values. All curves maintain linearity between 0.195 μM and 10 μM GFG.

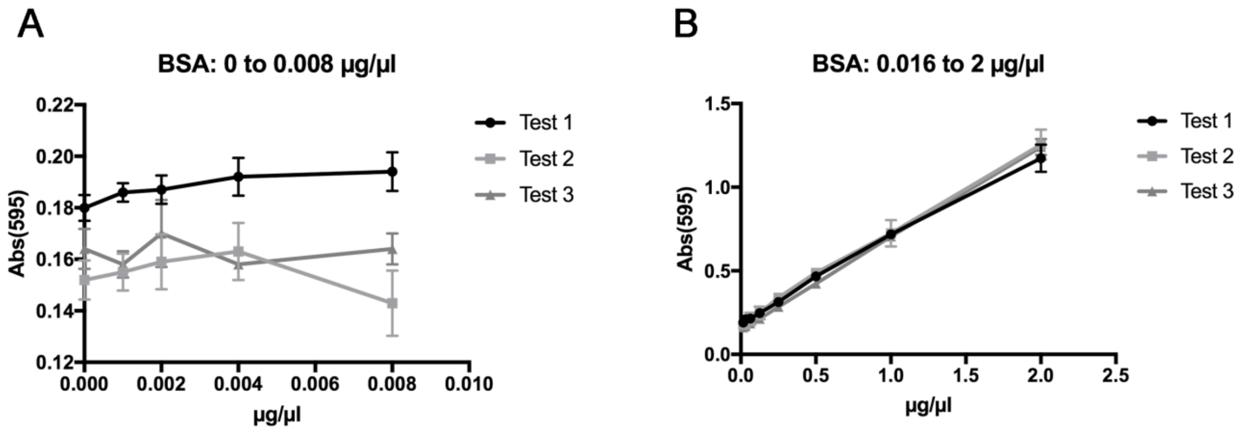

FIGURE 3S

Linearity range tested for proteins assay: correlation of proteins concentration with Abs_{595} using a bovine serum albumine (BSA) solution. Correlation is non linear below 0.008 µg/µl BSA (A); linearity ranges from 0.016 µg/µl to 2 µg/µl (B). Test was conducted three times (Test 1 to 3).

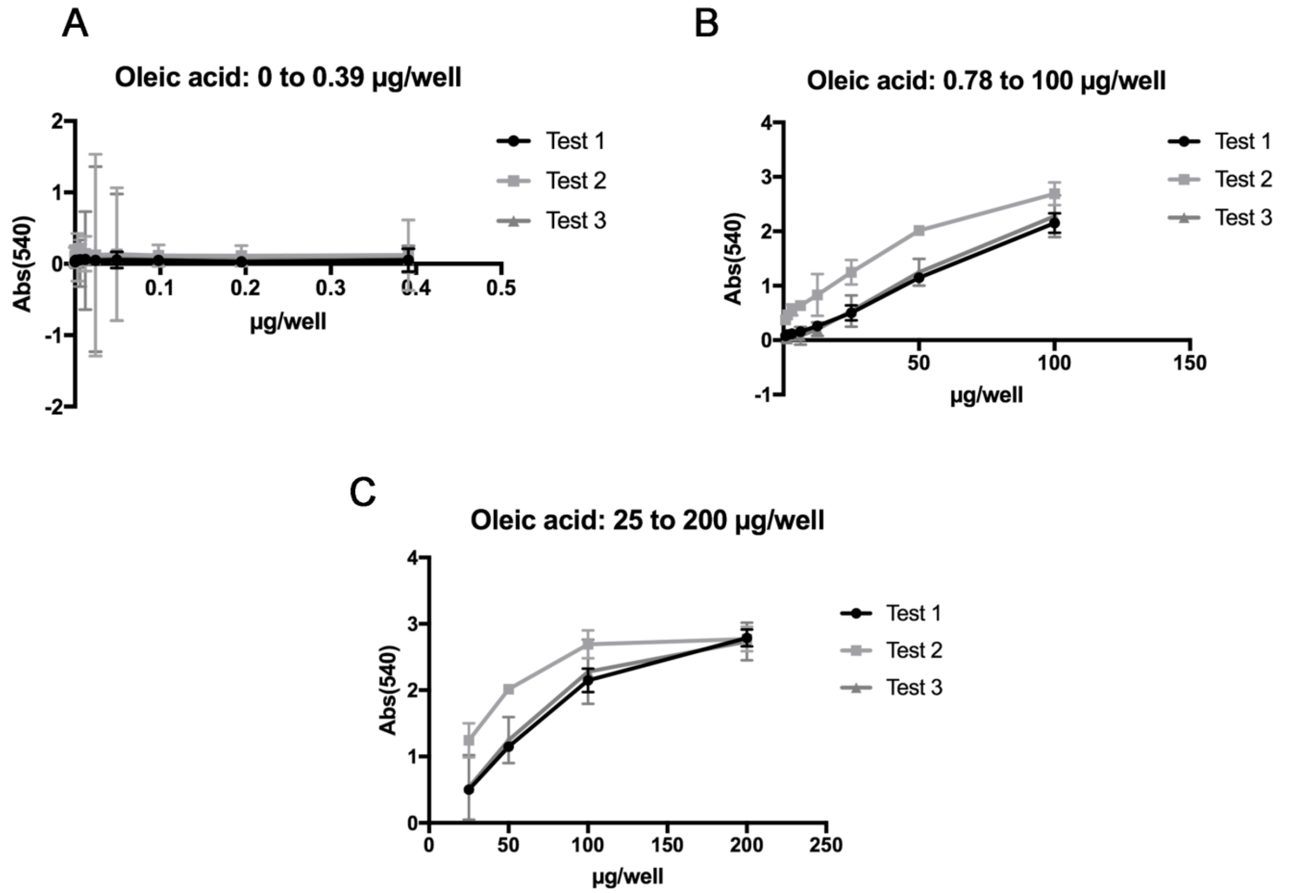

FIGURE 4S

Linearity range tested for lipids assay: correlation of lipids concentration with Abs_{540} using a oleic acid solution. Correlation is non linear below 0.39 µg/well oleic acid (A); linearity ranges from 0.78 µg/well to 100 µg/well (B). Above 100 µg/well, linearity is not maintained (C). Test was conducted three times (Test 1 to 3).

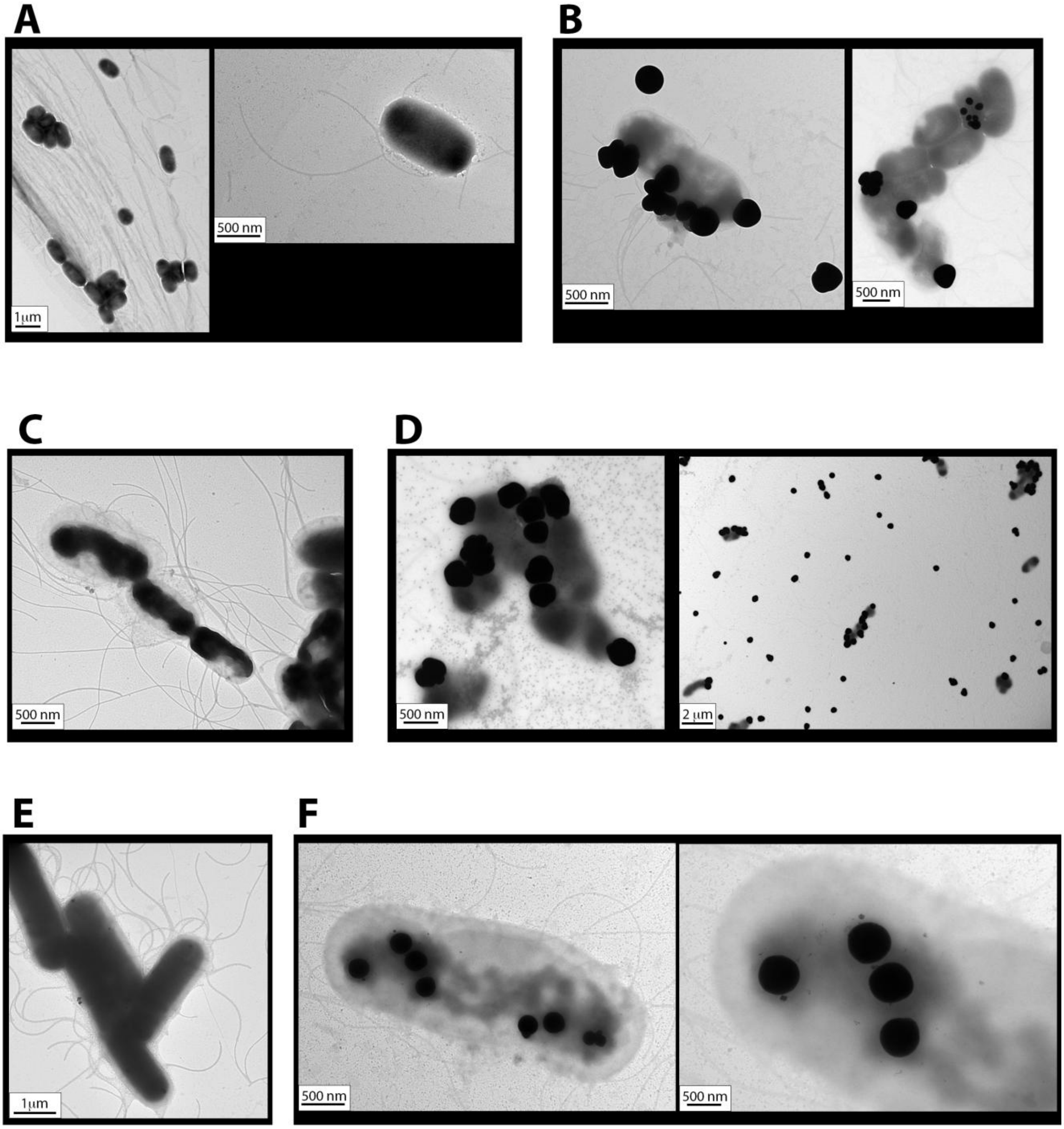

FIGURE 5S
Cultures of bacteria in Nutrient medium: R2A: control (A) and after 24 h exposition to selenite. R2D: control (C) and after 24 h exposition to selenite (D). R1E: control (E) and after 72 h exposition to selenite (D). SeNPs are clearly visible in all exposed cultures (B, D, F).

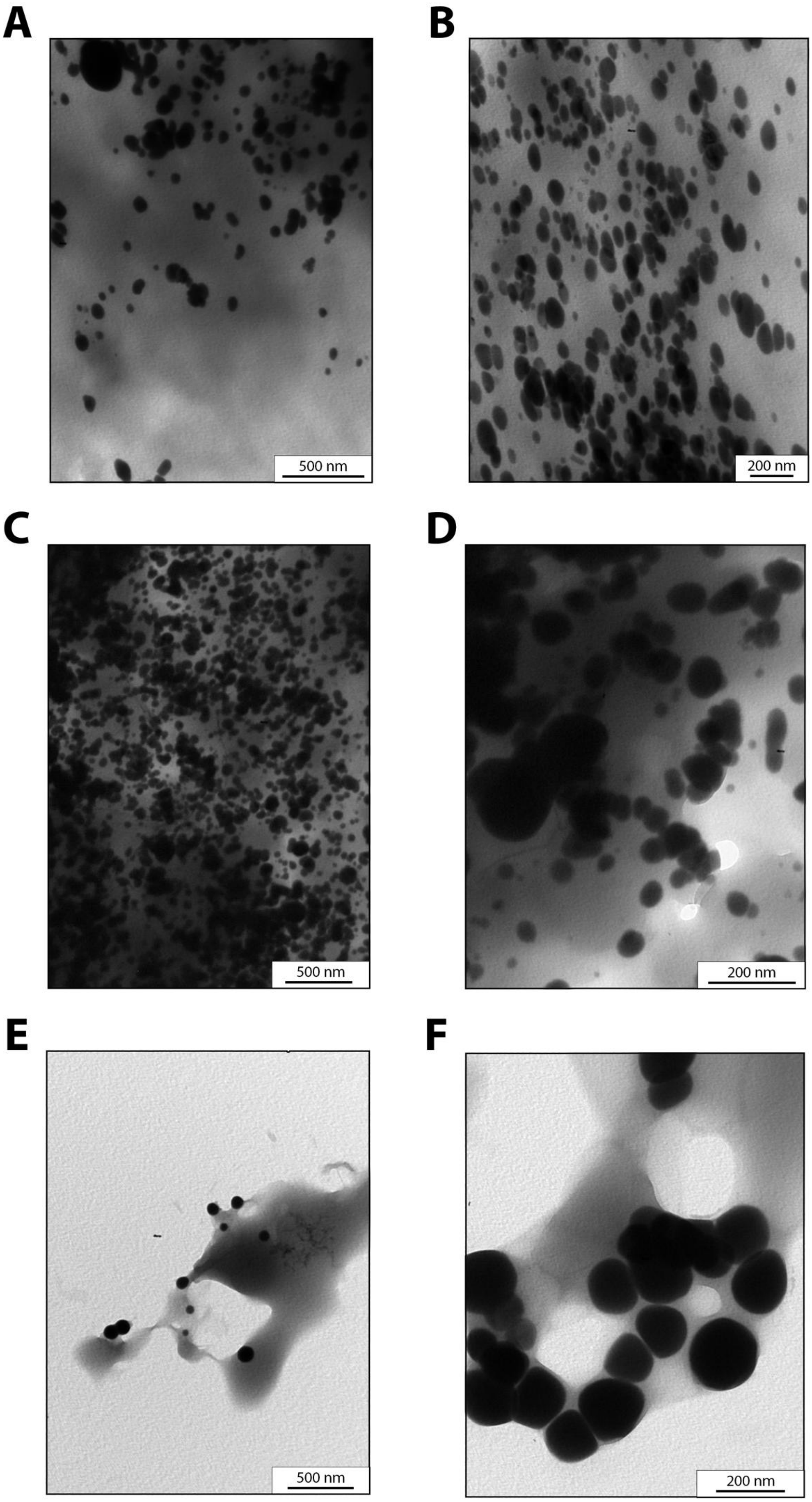
A
500 nm
B
200 nm
C
500 nm
D
200 nm
E
500 nm
F
200 nm

FIGURE 6S

SeNPs synthesized by *B. mycoides* SeITE01: A, B: standard sample. C, D: sample after detergent treatment with 2% Triton X-100, aggregates are visible as darker clusters. E, F: sample after detergent treatment with 10% SDS.

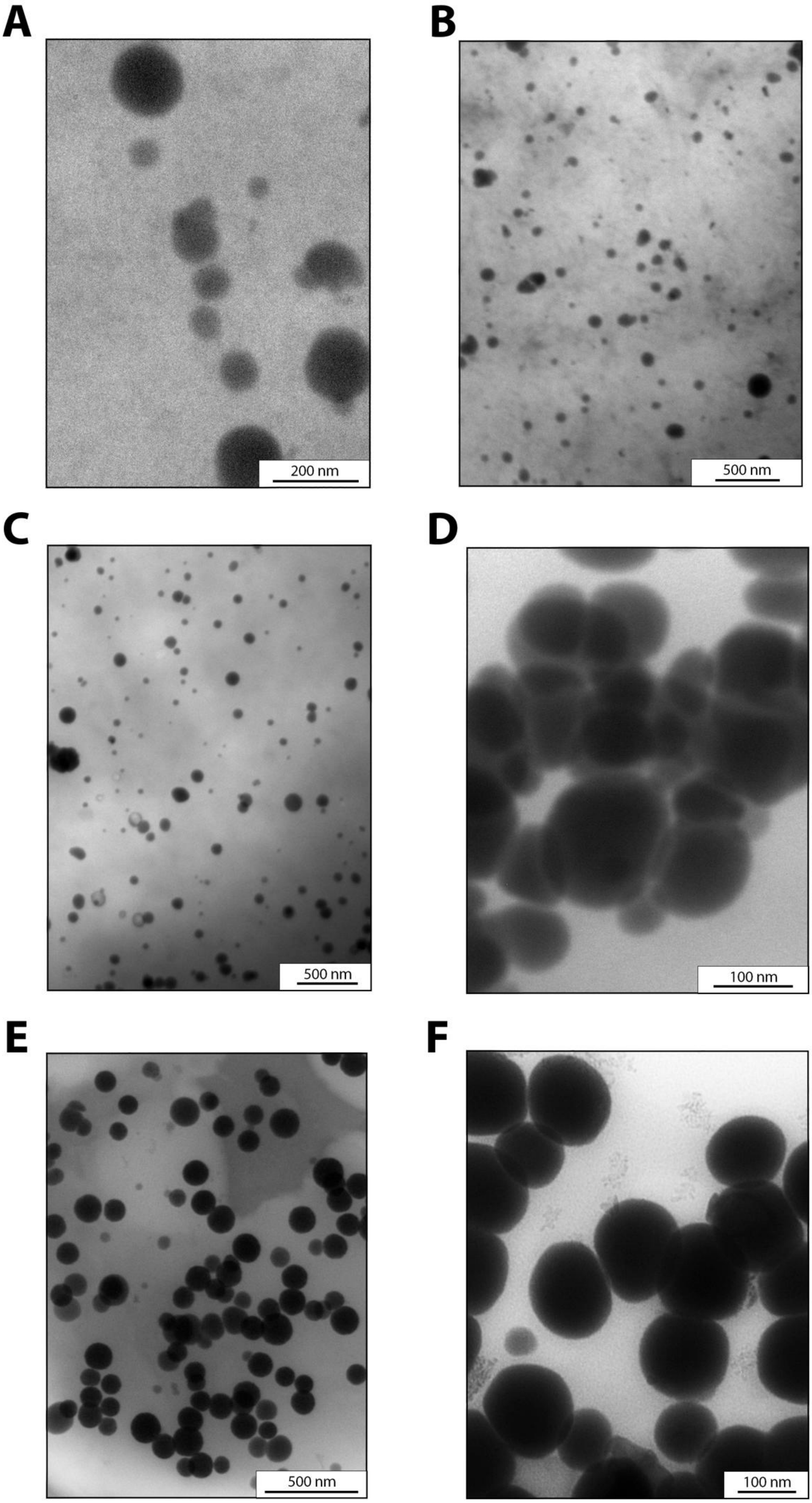
A
200 nm
B
500 nm
C
500 nm
D
100 nm
E
500 nm
F
100 nm

FIGURE 7S

SeNPs synthesized by *S. maltophilia* SeITE02. A, B: standard sample. C: sample after detergent treatment with 2% Triton X-100, here SeNPs have not aggregated. D: sample after detergent treatment with 2% Triton X-100: this is a representative figure of the aggregates which were visible throughout the sample; this correlates well with the measurement of a high Polydispersity (0.9). E, F: sample after detergent treatment with 10% SDS.
